# S-Nitroso Human Serum Albumin Enhances Left Ventricle Hemodynamic Performance and Reduces Myocardial Damage after Local Ischemia–Reperfusion Injury

**DOI:** 10.3390/biomedicines12071434

**Published:** 2024-06-27

**Authors:** Daniele Linardi, Seth Hallström, Giovanni Battista Luciani, Alessio Rungatscher

**Affiliations:** 1 Cardiac Surgery Department, University of Verona, 37129 Verona, Italy; daniele.linardi@aovr.veneto.it (D.L.);; 2 Division of Medicinal Chemistry, Otto Loewi Research Center, Medical University of Graz, 8010 Graz, Austria; seth.hallstroem@medunigraz.at; 3 Center for Biomedical Research and Translational Surgery, Medical University of Vienna, 1090 Vienna, Austria

**Keywords:** acute myocardial infarction, ischemia–reperfusion damage, nitric oxide, NO donor, nitrosylated albumin

## Abstract

Endothelial nitric oxide (NO) production is crucial in maintaining vascular homeostasis. However, in the context of ischemia–reperfusion (I/R) injury, uncoupled endothelial nitric oxide synthase (eNOS) can exacerbate reactive oxygen species (ROS) generation. Supplementation with S-nitroso human serum albumin (S-NO-HSA) offers a potential solution by mitigating eNOS uncoupling, thereby enhancing NO bioavailability. In a study conducted at the University of Verona, male rats underwent thoracotomy followed by 30 min left anterior descendant coronary (LAD) occlusion and subsequent reperfusion. Hemodynamic parameters were meticulously assessed using a conductance catheter inserted via the carotid artery. The rats were stratified into two main groups based on reperfusion duration and the timing of drug infusion, with the effects of S-NO-HSA evaluated after 2 or 24 h. Remarkably, intravenous administration of S-NO-HSA, initiated before or during ischemia, exhibited notable benefits. It significantly improved left ventricular function, safeguarded energetic substrates such as phosphocreatine and ATP, and sustained glutathione levels akin to basal conditions, indicative of diminished oxidative stress. The data from this study strongly suggest a protective role for S-NO-HSA in mitigating I/R injury induced by LAD artery occlusion, a phenomenon observed at both 2 and 24 h post-reperfusion. These findings underscore the promising therapeutic potential of NO supplementation in alleviating myocardial damage subsequent to ischemic insult.

## 1. Introduction

Acute myocardial infarction is best treated through timely coronary reperfusion, as it can reduce the infarct area, improve myocardial function, and increase the likelihood of survival. However, reperfusion injury may occur, leading to the deterioration of myocardial function. This injury is characterized by stunned myocardium, endothelial and cellular damage, or necrosis [1,2,3].

The causes of reperfusion injury are multifactorial and include the rapid production of oxygen free radicals (ROS) by endothelial cells, activated leukocytes, or even myocardial cells [4]. Endothelial cells play a crucial role in maintaining blood flow, preventing the coagulation process, and modulating vascular tone. When ischemia–reperfusion (I/R) damage occurs, the endothelium loses its quiescent state and becomes a highly permeable, prothrombotic, and proinflammatory tissue [5,6].

NO production influences endothelial function by vasodilating, inhibiting adhesion and platelet aggregation, maintaining restrictive diffusion, and inhibiting the adhesion of neutrophils [7,8,9]. Coronary ischemia–reperfusion dramatically suppresses basal NO production [10,11]. L-arginine, necessary for normal production of NO, decreases during ischemic damage and promotes the uncoupling of endothelial nitric oxide synthase (eNOS) and the formation of superoxide anion (O_2_^−^). The simultaneous presence of NO and O_2_^−^ triggers a reaction that leads to the formation of peroxynitrite (ONOO^−^), a highly cytotoxic component. Oxidative stress leads to enzymatic inactivation, DNA damage, lipid peroxidation, and membrane damage.

Nitrosothiols such as S-nitroso cysteine, S-nitroso glutathione, and nitrosylated albumin (S-NO-HSA) can release NO, exhibiting properties similar to NO. In contrast to low-molecular-weight S-nitrosothiols (S-nitroso cysteine and S-nitroso glutathione) with slightly enhanced half-lives compared to NO, S-NO-HSA has a high molecular weight and has, in comparison, a highly prolonged half-life of around 16 min in vivo. S-NO-HSA is produced by S-nitrosylation of human serum albumin after a preparation step that eliminates the mixed disulfides at Cys-34 and has been shown to have a long-lasting and slow NO release [12].

In a study involving treatment with S-NO-HSA in a rabbit hind limb I/R model, S-NO-HSA treatment preserved the function of eNOS, prevented its uncoupling, stabilized the basal NO production, decreased the production of oxidized species and, therefore, had beneficial effects in reducing I/R injury. The measurement of high-energy phosphates (HEP) also demonstrated greater preservation of mitochondrial function with S-NO-HSA treatment [13]. In further studies with global myocardial ischemia and reperfusion, the use of S-NO-HSA substantially increased cardiac output, diastolic function, and myocardial perfusion; it was also noted that the infusion of S-NO-HSA preserved the function of eNOS, stabilized NO production, and decreased the production of O_2_^−^ and ONOO^−^ [14,15,16].

Our study intended to test the effects of NO supplementation with continuous intravenous S-NO-HSA infusion in a model of local myocardial ischemia through left anterior descending artery (LAD) temporary ligation. We investigated the effects of S-NO-HSA on hemodynamic performance, the preservation of HEP, and a reduction in oxidative stress after local I/R injury at 2 h and 24 h of reperfusion.

## 2. Materials and Methods

### 2.1. Experimental Setting

The experimental investigation was carried out at the C.I.R.S.A.L. (Interdepartmental Center for Research on Laboratory Animals) of the Faculty of Medicine of the University of Verona. Adherence to prevailing regulations (Helsinki Declaration and “Guide to the Care and Use of Animals Laboratory-Institute of Animal Resources Laboratory-National Institutes of Health”) was ensured throughout the whole experimental protocol. Approval for the experimental protocol was granted by both the technical–scientific committee of the University of Verona and the ethical committee of the National Health Ministry under authorization 568/2020-PR.

Male Sprague Dawley rats, weighing 300 ± 50 g, were housed in environments maintaining controlled temperature and humidity, adhering to a standard day–night cycle. Their diet consisted of standard feed, and they had access to water ad libitum.

Anesthesia induction and maintenance were achieved with sevoflurane (Sevorane Abbot, Baar, Switzerland) at 5% for induction, followed by 1.5% after orotracheal intubation using an 18 G venous cannula. Analgesia was sustained with subcutaneous administration of ketoprofen (1 mg/kg). Mechanical ventilation was administered using a rodent-specific mechanical respirator (Harvard Apparatus Inspira asv, Holliston, MA, USA), delivering a mixture of oxygen and sevoflurane with a fraction of inspired oxygen of 90%, a tidal volume of 10 mL/kg, and a frequency of 80 breaths per minute. Surgical conditions were maintained with room temperature between 23 and 25 °C, insulation between the animal and the operating table using a cork panel, and an infrared lamp ensuring the animals’ thermoregulation.

The surgical procedure involved shaving the thoracic and ventral surfaces of the neck and hind leg, followed by skin disinfection using chlorhexidine. Subsequently, an incision was made along the median line of the neck, allowing for the isolation of the right carotid artery. A miniaturized 2-Fr catheter (PV catheter model SPR 838, Millar Instruments, Houston, TX, USA) was then inserted into the carotid and advanced to the left ventricle. This catheter was connected to a Power-Lab unit (AD Instruments, Colorado Springs, CO, USA) and a computer, facilitating the real-time display of pressure–volume curves and data recording using Chart software (Lab Chart 7, AD Instruments).

### 2.2. Surgical Procedure

**Figure 1:**
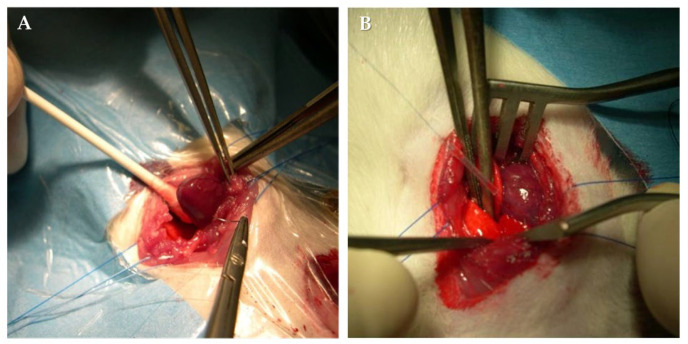
LAD ligation with 6.0 stitch (**A**) and temporary occlusion (ischemic time 30 min) with a tourniquet (**B**).

A left thoracotomy procedure was performed between the fourth and fifth intercostal space, and the pericardium was opened, exposing the heart. At about 2 mm from the apex of the left auricle, LAD was surrounded by a polypropylene 6-0 suture (Prolene, Ethicon, J&J Medical Device, New Brunswick, NJ, USA). The ends of the suture were passed through a small polyethylene tube (18 G), narrowed against the epicardium, and clamped; a complete occlusion of the vessel was guaranteed for 30 min. The immediate development of a well-demarcated area of pallor, which was distinguished from the perfused myocardium for dyskinesia, was always evident in occlusion. After 30 min, the polyethylene tube was removed to allow myocardial reperfusion (Figure 1).

We identified the ischemic area at risk (AR) in a preliminary study using LAD occlusion. The ischemic area was demarcated in negative by staining with the Evans Blue solution and had an average weight of 0.220 + 0.014 g. The mass of the ischemic area at risk in relation to that of the left ventricle (AR/LV) was 40.2% + 2.7%, while the necrotic area mass in relation to the ischemic area at risk (IF/AR) on average was 65.2% + 3.4%. There were no significant differences between different reperfusion times in the different groups (Table 1).

### 2.3. S-NO-HSA Preparation

S-NO-HSA preparation was conducted as already described in a previous paper [13] (p. 2). Briefly, HSA underwent processing to achieve a maximal presence of free thiol groups at position Cys-34. Prior to nitrosation, intermolecular disulfides were disassembled. The initial material (20% HSA; Baxter, Deerfield, IL, USA) underwent reduction using mercaptoethanol (at a 10 to 20-fold molar excess) in a buffer solution (mmol/L: sodium phosphate 1, ethylenediaminetetraacetic acid 2, and sodium chloride 150 adjusted to pH = 6.0–6.2 with hydrochloric acid (HCl)). This process occurred over 12 to 48 h at 4 °C under a nitrogen atmosphere, followed by purification through gel permeation chromatography (TSK-HW40F; using H_2_O as the mobile phase). Thiol nitrosylation was carried out using sodium nitrite at a ratio ranging from 1:1 to 1:1.5 of freely available thiol groups to nitrite in 0.2 mol/L HCl (pH = 1.5–2.5) for 30 min at 25 °C. Subsequently, the mixture was neutralized with 1 mol/L sodium hydroxide, and S-NO-HSA was purified once more via gel permeation chromatography (TSK-HW40F; using H_2_O as the mobile phase) and then subjected to lyophilization. The resulting S-NO-HSA was dissolved, and HSA was diluted with a solution of 0.9% saline before being continuously infused via a 24 G catheter into the femoral vein. S-NO-HSA was infused in the different groups at 0.2 µmol/kg/h; in blood, S-NO-HSA decomposes in NO and HSA and has an initial half-life of 16 min determined after the stop of infusion. The release of NO from S-NO-HSA due to its half-life after the stop of infusion at this dose is only relevant for approximately 45 min.

### 2.4. Experimental Protocol

**Figure 2:**
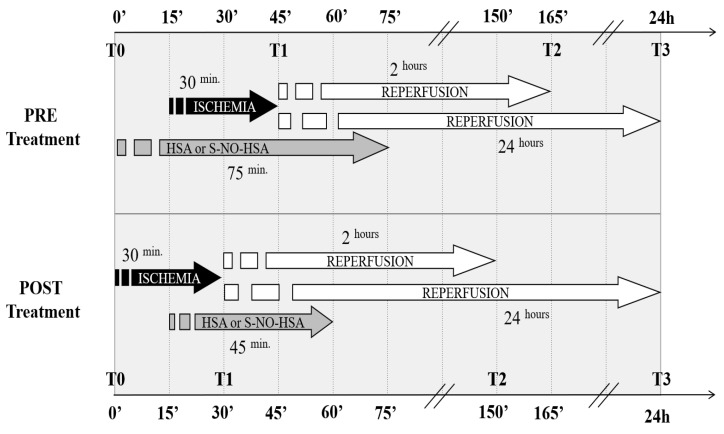
Experimental protocol of the study. Rats were divided into two main groups: one received pretreatment with HSA or SNOHSA 15 min before ischemia induction; the other group received post-treatment with HSA or SNOHSA 15 min after ischemia induction.

Animals were randomly allocated into two primary groups, each comprising 20 animals. Within each group, 10 rats were administered the experimental drug S-NO-HSA, while the other 10 received HSA as a control. Notably, the treatment timing varied between the two groups: In the first group, drug infusion commenced 15 min prior to ischemia (pretreatment), continued throughout the ischemic period, and concluded 30 min post-ischemia, resulting in a total drug infusion duration of 75 min (referred to as pre-HSA or pre-S-NO-HSA). Conversely, in the second group, drug infusion commenced 15 min after the onset of ischemia (post-treatment) and persisted for 30 min post-ischemia, totaling 45 min of drug infusion (referred to as post-HSA or post-S-NO-HSA). It is crucial to note that the timing of drug administration has consequences: In the post-treatment group, the drug could only reach the infarcted ventricular area following reperfusion. Conversely, in the pretreatment group, S-NO-HSA was administered 15 min before coronary occlusion, allowing it to act on the area at risk of ischemia prior to occlusion (Figure 2).

During the experiments, ischemia was induced for 30 min by ligation of the LAD. The administration of S-NO-HSA and HSA for control animals occurred via the femoral vein, using an elastomeric pump at 0.2 µmol/kg/h. The experiment was repeated when fatal or non-reversible arrhythmias occurred during ischemia or the initial reperfusion phase. Notably, no significant difference in the incidence of fatal arrhythmias was observed between the groups.

Following ischemia–reperfusion injury, the two main groups underwent distinct reperfusion strategies. Half of the rats were observed for two hours, after which hemodynamic assessments were conducted using a Millar catheter. Subsequently, these rats were sacrificed, and myocardial tissue samples were collected for biochemical analysis. The remaining half of the rats were extubated, allowed to awaken, and repositioned in cages. After 24 h, these rats were once again anesthetized, intubated, connected to a respirator, and subjected to hemodynamic studies before being sacrificed. Myocardial tissue samples were then obtained for further analysis.

To both groups, a sham group was added where the rats were anesthetized and intubated; then, a left thoracotomy was performed, and the pericardium was opened, but no ischemia or drug infusion was performed.

In summary, a total of 50 rats were utilized for this study, 5 rats for each subgroup, namely HSA with 2 h reperfusion, HSA with 24 h reperfusion, S-NO-HSA with 2 h reperfusion, and S-NO-HSA with 24 h reperfusion (pretreatment and post-treatment), and 10 rats were used for the sham group (5 with 2 h of observation and 5 with 24 h observation).

### 2.5. Hemodynamic Analysis

Hemodynamic data from the left ventricle were obtained using a Millar catheter during temporary occlusion of the inferior vena cava at four distinct time points: At the onset of the experiment under basal conditions (T0), immediately prior to removal of the LAD ligature following 30 min of myocardial ischemia (T1), after 2 h of reperfusion (T2), and following 24 h of reperfusion (T3) (Figure 2).

The recorded hemodynamic parameters portray systolic and diastolic left ventricular function. Systolic function: ejection fraction (EF), stroke volume (SV), maximum increase in systolic pressure (dp/dt max), maximal power (max PWR), and preload adjusted maximal power (PAMP). Diastolic function: left ventricular end-diastolic pressure (LVEDP) maximum decrease in diastolic pressure (dp/dt min), and the tau-Weiss time constant (tau-Weiss). 

LVEDP, SV, and EF are well-known hemodynamic parameters used in clinical practice. Maximal power indicates power generated by the left ventricle at ejection time. It can be measured by the product of the ventricular pressure and the volume change rate or, alternatively, the flow in the aorta. Max PWR and max PWR divided by the square of the end-diastolic volume, generally known as preload adjusted maximal PWR (PAMP), are both used to reflect changes in left ventricle contractile status rather independently of the afterload. However, max PWR is highly sensitive to changes in preload and was, therefore, partially abandoned in favor of PAMP. Parameters dp/dt min and dp/dt max represent the ventricle’s minimum and maximum rate of pressure change. Dp/dt max (peak dp/dt) is one of the oldest measures of left ventricular global contractility and is a good index of ventricular performance that is not influenced by afterload, wall motion abnormalities, or variations in ventricular anatomy and morphology. Dp/dt max occurs during systolic contraction when the rate of change in pressure over time (slope of the curve) achieves its peak value. An increase in contractility is manifested as an increase in dp/dt max during isovolumic contraction. Likewise, an increase in diastolic function or relaxation (lusitropy) causes increased dp/dt min during isovolumic relaxation. Hence, dp/dt min has been used as a valuable tool in analyzing isovolumic relaxation. 

Tau-Weiss measurement represents the exponential decay of the ventricular pressure during isovolumic relaxation. Several studies have shown that tau is a preload-independent measure of isovolumic relaxation.

### 2.6. Biochemical Analysis

#### 2.6.1. High-Energy Phosphates

A snap-frozen sample was obtained from the left ventricle region affected by ischemia–reperfusion injury at the conclusion of the designated reperfusion period. In particular, the tip of the freeze-clamp tong was precooled in liquid nitrogen before taking the biopsies, and after that, the samples were stored at −80 °C until further analysis.

The sample preparation and high-performance liquid chromatography (HPLC) measurement of ATP, ADP, AMP, and phosphocreatine were carried out as previously described [17,18]. A frozen tissue sample (50–100 mg) underwent homogenization using a microdismembranator and deproteinization with 500 µL of 0.4 mol/L perchloric acid. Following centrifugation (12,000× *g*), 150 µL of the acid extract was neutralized using 15–20 µL of 2 mol/L potassium carbonate at 4 °C. The resulting supernatant (20 µL injection volume), obtained post-centrifugation, was subjected to HPLC analysis. Separation was conducted on a Hypersil ODS column (5 µm; 250 × 4 mm I.D.) utilizing an L-2200 autosampler, two L-2130 HTA pumps, and an L-2450 diode array detector (all: VWR Hitachi, VWR, Vienna, Austria). Detector signals (absorbance at 214 and 254 nm) were recorded, and data requisition and analysis were performed using the EZchrom Elite program. The energy charge was determined using the following formula: (ATP + 0.5 ADP)/(AMP + ADP + ATP).

#### 2.6.2. Reduced and Oxidized Glutathione

For the determination of reduced and oxidized glutathione (GSH; GSSG), the analyses were also performed in principle according to previously described methods [19]. A portion of the supernatant obtained from the acid extract (0.4 mol/L perchloric acid) following centrifugation was utilized for HPLC analysis. Samples (10–20 µL injection volume) underwent chromatography on a Spherisorb S3ODS-2 column (3 µm, 125 mm × 4 mm I.D.) employing a SIL-20AC HT autosampler with a CBM-20A communication bus module (Shimadzu), a 582 HPLC pump (ESA), a pulsation damper (Shodex, model DPI), and a Coulochem II electrochemical detector (ESA) equipped with a 5020 guard cell (potential: 0.4 V) and a 5011 analytical cell (first electrode potential: 0.5 V; second analytical electrode potential: 0.90—0.95 V, for GSH and GSSG). Detector signals were acquired via an analog interface (SS 420x, Scientific Software Inc., Lewes, UK) connected to a personal computer, with data acquisition and analysis performed using the EZchrom Elite program. The mobile phase comprised 0.1 mol/L sodium acetate, 0.1 mol/L sodium dihydrogen phosphate, 400 mg/L sodium dodecyl sulfate adjusted to pH = 2.0, and 2% acetonitrile (*v*/*v*), flowing at a rate of 1 mL/min.

The pellets from the acid extract were dissolved in 1 mL of 0.1 mol/L sodium hydroxide and then further diluted 1:10 with physiological saline for protein determination using the BCA Protein Assay (Pierce; Pierce Biotechnology, Rockford, IL, USA).

### 2.7. Statistical Analysis

For the hemodynamic and biochemical analysis on HEP and GSSG/GSH, a one-way analysis of variance for between-group comparisons, followed by an unpaired two-tailed Student’s *t*-test to evaluate differences between groups was used. A paired *t*-test was used to evaluate differences between groups and baseline. The results in the graphs are presented as means ± standard error. Values were considered significant with *p* < 0.05.

## 3. Results

### 3.1. Hemodynamic Analysis

Hemodynamic data of the left ventricle during the different time points were analyzed. At T0, hemodynamic parameters reflected identical conditions across all analyzed groups (sham, pre-HSA, pre-S-NO-HSA, post-HSA, and post-S-NO-HSA), yielding no significant differences, as anticipated.

At T1, after 30 min of ischemia just before ligature removal, notable differences emerged between the two primary groups, exerting differences between pretreatment and post-treatment with S-NO-HSA.

**Figure 3:**
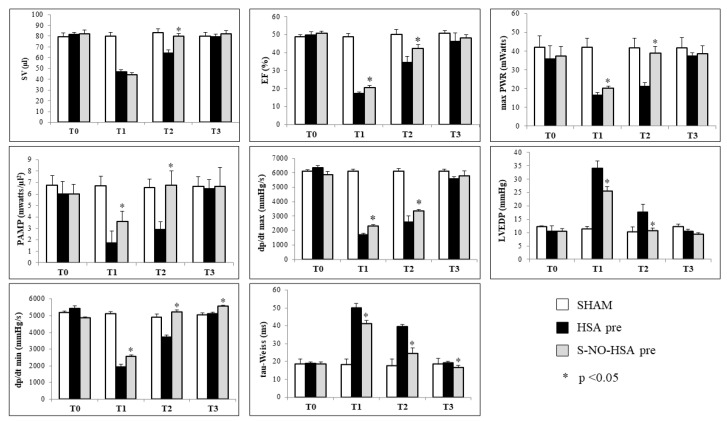
Left ventricle hemodynamic parameters in pretreated rats with HSA or S-NO-HSA at different time points (T0, basal; T1, ischemia; T2, 2 h of reperfusion; T3, 24 h of reperfusion); * *p* < 0.05 pre-S-NO-HSA vs. pre-HSA. SV = stroke volume; EF = ejection fraction; max PWR = maximal power; PAMP = preload adjusted maximal power; dP/dt max = maximum increase in systolic pressure; LVEDP = left ventricular end-diastolic pressure; dP/dt min = maximum decrease in diastolic pressure; tau-Weiss = tau-Weiss time constant.

In the pretreatment group, significant differences (*p* < 0.05) were observed between treated (pre-S-NO-HSA) and control group rats (pre-HSA) during ischemia. Parameters indicative of both systolic function (EF, max Pwr, PAMP, dp/dt max) and diastolic function (LVEDP, dp/dt min, tau-Weiss) were affected, with improvements noted upon the initiation of S-NO-HSA infusion 15 min prior to LAD ligature (pre-S-NO-HSA vs. pre-HSA: EF (%) 44.31 ± 1.72 vs. 46.73 ± 2.21; PAMP (mW/µL^2^) 3.63 ± 0.89 vs. 1.72 ±1.04; dp/dt max (mmHg/s) 2321 ± 97 vs. 1696 ± 125; LVEDP (mmHg) 25.50 ± 1.70 vs. 34.10 ± 2.72 tau-Weiss (ms) 41.21 ± 1.70 vs. 50.02 ± 2.50; dp/dt min (mmHg/s) 2575 ± 75 vs. 1925 ± 157; *p* < 0.05). Moreover, all analyzed parameters demonstrated significant improvement in rats treated with S-NO-HSA following 2 h of reperfusion (T2), with S-NO-HSA exerting a preservative effect on both systolic and diastolic function, aligning hemodynamic parameters closely with basal values (pre-S-NO-HSA vs. pre-HSA vs. sham: SV (µL) 80.22 ± 1.72 vs. 64.35 ± 3.01 vs. 83.11 ± 3.54; EF (%) 42.40 ± 1.90 vs. 34.60 ± 3.20 vs. 50.10 ± 2.60; PAMP (mW/µL^2^) 6.79 ± 1.22 vs. 2.88 ± 0.69 vs. 6.54 ± 0.78; dp/dt max (mmHg/s) 3367 ± 98 vs. 2584 ± 434 vs. 6122 ± 155; LVEDP (mmHg) 10.67 ± 0.98 vs. 17.61 ± 3.04 vs. 10.24 ± 1.87; tau-Weiss (ms) 24.43 ± 3.21 vs. 39.68 ± 0.89 vs. 17.63 ± 3.88; dp/dt min (mmHg/s) 5241 ± 115 vs. 3711 ± 120 vs. 4922 ± 179; *p* < 0.05). By T3, systolic and diastolic function values were comparable between treated (pre-S-NO-HSA) and untreated (pre-HSA) rats, with both groups exhibiting values similar to those of sham-operated rats and, therefore, similar to basal values (Figure 3).

**Figure 4:**
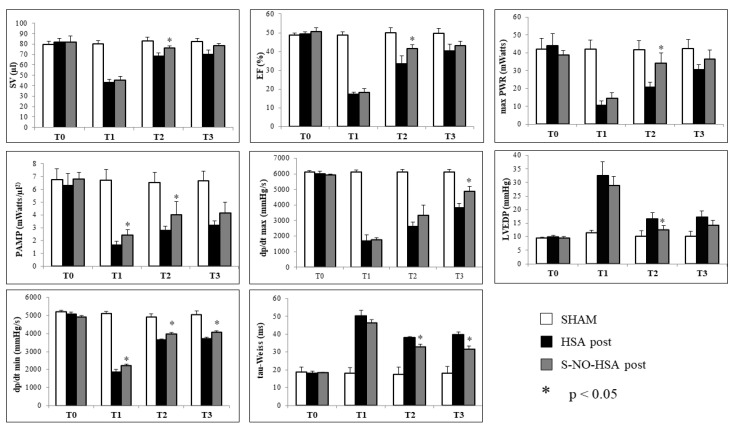
Left ventricle hemodynamic parameters in post-treated rats with HSA or S-NO-HSA at different time points (T0, basal; T1, ischemia; T2, 2 h of reperfusion; T3, 24 h of reperfusion). * *p* < 0.05 post-S-NO-HSA vs. post-HSA. SV = stroke volume; EF = ejection fraction; max PWR = maximal power; PAMP = preload adjusted maximal power; dP/dt max = maximum increase in systolic pressure; LVEDP = left ventricular end-diastolic pressure; dP/dt min = maximum decrease in diastolic pressure; tau-Weiss = tau-Weiss time constant.

In the post-treatment group (post-HSA and post-S-NO-HSA), during the ischemic phase (T1), significant differences (*p* < 0.05) between treated (post-S-NO-HSA) and untreated (post-HSA) rats were observed solely in preload adjusted maximal power and dp/dt min (post-S-NO-HSA vs. post-HSA: PAMP (mW/µL^2^) 2.45 ± 0.43 vs. 1.65 ± 0.32; dp/dt min (mmHg/s) 2221 ± 87 vs. 1883 ± 125; *p* < 0.05). However, following 2 h of reperfusion (T2) with drug administration continuing after tourniquet release, all analyzed parameters, except dp/dt max (3330 ± 674 vs. 2640 ± 280 (mmHg/s); *p* > 0.05), exhibited significant improvement in S-NO-HSA-treated rats compared to those receiving HSA. Positive effects on left ventricular function were still evident after 24 h of reperfusion. Parameters such as dp/dt max, as well as tau-Weiss and dp/dt min, markers of diastolic function, aligned more closely with sham and basal level values in rats treated with S-NO-HSA (post-S-NO-HSA vs. post-HSA vs. sham: dp/dt max (mmHg/s) 4858 ± 355 vs. 3841 ± 276 vs. 6125 ± 157; dp/dt min (mmHg/s) 4066 ± 94 vs. 3705 ± 104 vs. 5086 ± 190; tau-Weiss (ms) 31.65 ± 1.89 vs. 39.84 ± 1.46 vs. 18.03 ± 4.02; *p* < 0.05). In both pretreated and post-treated groups, the administration of S-NO-HSA demonstrates beneficial effects on the left ventricle’s systolic and diastolic hemodynamic function, in particular after 2 h of reperfusion (Figure 4).

**Figure 5:**
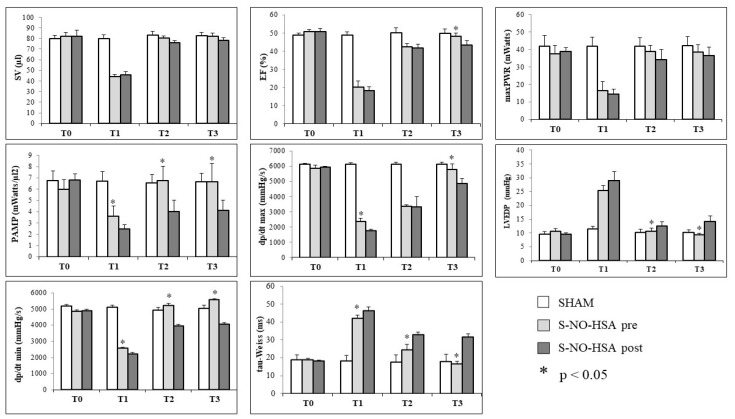
Left ventricle hemodynamic parameters pretreated S-NO-HSA and post-treated S-NO-HSA at different time points (T0, basal; T1, ischemia; T2, 2 h of reperfusion; T3, 24 h of reperfusion); * *p* < 0.05 pre-S-NO-HSA vs. post-S-NO-HSA. SV = stroke volume; EF = ejection fraction; max PWR = maximal power; PAMP = preload adjusted maximal power; dP/dt max = maximum increase in systolic pressure; LVEDP = left ventricular end-diastolic pressure; dP/dt min = maximum decrease in diastolic pressure; tau-Weiss = tau-Weiss time constant.

A comparative analysis between the two treated groups reveals that S-NO-HSA pretreatment benefits both systolic and diastolic left ventricular function, evident even during the ischemic phase (T1), with superior values compared to S-NO-HSA post-treatment (pre-S-NO-HSA T1 vs. post-S-NO-HSA T1: PAMP (mW/µL^2^) 3.63 ± 0.89 vs. 2.45 ± 0.43; dp/dt max (mmHg/s) 2366 ± 217 vs. 1744 ± 129; LVEDP (mmHg) 25.50 ± 1.70 vs. 28.90 ± 3.28; tau-Weiss (ms) 42.18 ± 1.73 vs. 46.22 ± 2.11; dp/dt min (mmHg/s) 2575 ± 75 vs. 2221 ± 87.35; *p* < 0.05). Moreover, after two and twenty-four hours of reperfusion, pretreated and post-treated rats exhibit improved hemodynamic status. However, pretreatment appears more effective than post-treatment, showcasing a faster onset and sustained positive impact on left ventricular hemodynamic performance (pre-S-NO-HSA T2 vs. post-S-NO-HSA T2: PAMP (mW/µL^2^) 6.79 ± 1.22 vs. 4.02 ± 1.02; LVEDP (mmHg) 10.67 ± 0.98 vs. 12.54 ± 1.57; tau-Weiss 24.43 ± 3.21 vs. 32.88 ± 1.54; dp/dt min (mmHg/s) 5240 ± 115 vs. 3976 ± 74; *p* < 0.05) (pre-S-NO-HSA T3 vs. post-S-NO-HSA T3: EF (%) 48.20 ± 1.80 vs. 43.23 ± 2.4; PAMP (mW/µL^2^) 6.68 ± 1.63 vs. 4.15 ± 0.86; dp/dt max (mmHg/s) 5773 ± 373 vs. 4857 ± 355; LVEDP (mmHg) 9.35 ± 0.54 vs. 14.2 ± 1.89; tau-Weiss (ms) 16.56 ± 1.43 vs. 31.65 ± 1.89; dp/dt min (mmHg/s) 5578 ± 48 vs. 4066 ± 94; *p* < 0.05) (Figure 5).

### 3.2. High-Energy Phosphates and Energy Charge

**Figure 6:**
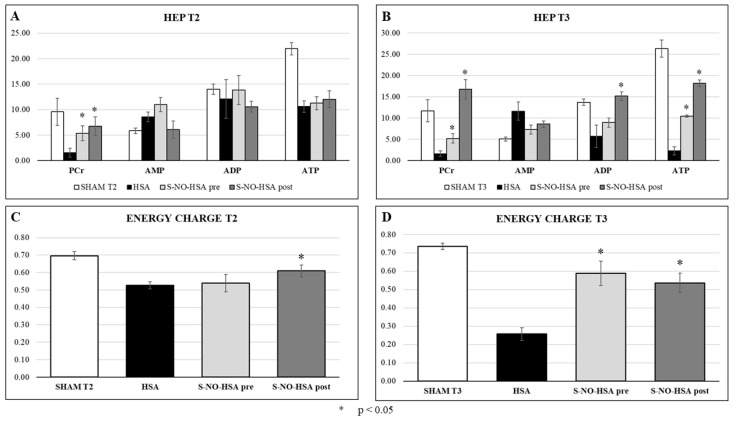
High-energy phosphates in biopsies of the left ventricle in the treatment group receiving pre-S-NO-HSA and post-S-NO-HSA compared with the control group (HSA) in two different time points: T2 (2 h reperfusion) and T3 (24 h reperfusion). Changes in PCr and adenine nucleotide levels (**A**,**B**) and energy charge (**C**,**D**) in S-NO-HSA vs. control group; * *p* < 0.05 vs. HSA.

Phosphocreatine (PCr), the buffering energy source for ATP in situations of energy demand, was significantly higher in S-NO-HSA-treated animals vs. HSA after 2 h of reperfusion (5.35 ± 1.02 pre-S-NO-HSA T2 vs. 6.74 ± 1.24 post-S-NO-HSA T2 vs. 1.54 ± 0.54 HSA T2; *p* < 0.05). The concentration of PCr and the difference to the control group was higher in post-treated than in pretreated rats and was higher at T3 rather than T2 (5.21 ± 0.74 pre-S-NO-HSA T3 vs. 16.78 ± 2.04 post-S-NO-HSA T3 vs. 1.59 ± 0.47 HSA T3) (Figure 6 Panel A and B).

After 2 h of reperfusion (T2), the energy charge [EC = (ATP + ADP)/(AMP + ADP + ATP)] was significantly higher in the post-S-NO-HSA group, but no significant differences were found in the pretreatment group (0.54 ± 0.05 pre-S-NO-HSA vs. 0.61 ± 0.03 post-S-NO-HSA vs. 0.53 ± 0.02 HSA).

After 24 h, energy charge was significantly higher in both S-NO-HSA-treated groups compared to control (0.59 ± 0.02 pre-S-NO-HSA T3 vs. 0.54 ± 0.13 post-S-NO-HSA T3 vs. 0.26 ± 0.04 HSA T3; *p* < 0.05) (Figure 6 Panel C and D).

### 3.3. Oxidative Stress

**Figure 7:**
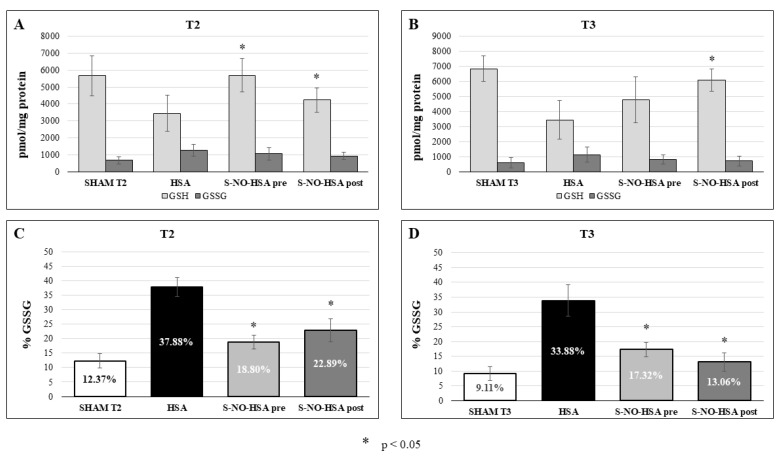
GSH and GSSG content in biopsies of the left ventricle in the treatment group receiving pre-S-NO-HSA and post-S-NO-HSA compared with the control group (HSA) at two different time points: T2 (2 h reperfusion) and T3 (24 h reperfusion). Changes in GSSG/GSH content (**A**,**B**) and the percentage of oxidized glutathione (GSSG) are illustrated (**C**,**D**); * *p* < 0.05 vs. HSA.

Utilizing the perchloride acid extracts obtained from the snap-frozen tissue of the left ventricle region affected by ischemia–reperfusion injury at 2 h and 24 h post-reperfusion, we determined the GSH and GSSG content. The results are illustrated in Figure 7 (Panel A and B). Notably, in rats treated with S-NO-HSA, the GSH contents within the left ventricular wall following ischemia–reperfusion injury were significantly higher compared to the control group. After 2 h, the GSH content was 3450 ± 1055 pmol/mg protein in the HSA-treated group, 5686 ± 989 pmol/mg protein in the S-NO-HSA-pretreated group, and 4229 ± 717 pmol/mg protein in the S-NO-HSA-post-treated group, with a significant difference in both the groups treated with S-NO-HSA (*p* < 0.05).

After 24 h, the GSH content was 3450 ± 1281 pmol/mg protein in the HSA-treated group, 4779 ± 1516 pmol/mg protein in the S-NO-HSA-pretreated group, and 6112 ± 741 pmol/mg protein in the S-NO-HSA-post-treated group, with a significant difference in the post-treated group (*p* < 0.05). In accordance with this, the percentage of GSSG significantly increased in the untreated HSA group and was reduced by S-NO-HSA treatment at T2 and T3 (Figure 7, Panel C and D).

## 4. Discussion

The NO produced by endothelium exerts multiple functions on the cardiocirculatory system. Not only does it regulate the tone of vascular smooth muscle and, therefore, peripheral resistance, but it also has antithrombotic effects by inhibiting platelet aggregation and adhesion and anti-inflammatory effects by inhibiting neutrophil activation and the synthesis of proinflammatory cytokines. Some studies have shown that the administration of NO-donor molecules or the induction of NO synthesis can prevent myocardial I/R injury. On the other hand, there is evidence that the pharmacological inhibition of NO production can also reduce infarct size; the synthesis of excessive amounts of NO can exert adverse effects, promoting ONOO^−^ formation during reperfusion [20,21,22,23]. Other Studies have highlighted NO protective effects with several potential mechanisms: maintenance of the correct function of the endothelium; a reduction in cellular calcium overload by inhibiting the inositol 1,4,5-triphosphate and membrane channels; a reduction in myocardial oxygen consumption by regulating mitochondrial function; and the inhibition of apoptosis by caspases cysteine nitrosylation [24,25,26,27,28]. 

We hypothesize that constitutive eNOS plays a pivotal role in the onset and progression of I/R injury. Ischemia initiates an upsurge in intracellular calcium ion levels, primarily mediated by heightened catecholamine concentrations. This surge in calcium ions prompts an abnormal activation of eNOS, leading to excessive NO generation and subsequently instigating the production of reactive oxygen species and cytotoxic compounds. While the mechanisms driving endothelial dysfunction are multifaceted, it is imperative to recognize that the augmented generation of oxygen-derived free radicals by a dysfunctional eNOS significantly contributes to this pathological process. Consequently, our approach to employing S-NO-HSA in a model of 30 min LAD occlusion relies on a strategic framework. Firstly, the gradual release of NO by S-NO-HSA actively counteracts endothelial dysfunction by impeding eNOS uncoupling. The exogenous NO liberated by S-NO-HSA leads to the feedback inhibition of eNOS and therefore reduces its NO production and diminishes the turnover rate of eNOS substrates and cofactors. As a consequence, eNOS uncoupling is prevented or minimized, and O_2_^−^ as well as cytotoxic ONOO^−^ production is reduced, thereby ameliorating oxidative stress-induced damage [14] (p. 2). The early administration of NO donors by systemic or local administration during the early stages of ischemia–reperfusion injury could recreate a physiological equilibrium condition with adequate initial NO concentrations. From our point of view, recognizing that NO has a protective role and that overproduction due to iNOS may have a possibly harmful effect requires a detailed understanding concerning new therapeutic strategies for the myocardium and other vital organs [29,30].

In this study, we measured the effect of NO administration via S-NO-HSA on the left ventricle in a model of standardized coronary occlusion with treatment applied before or after the onset of ischemia. The two groups (pretreatment and post-treatment) can be compared to two clinical scenarios: treatment of a coronary occlusion after ischemia onset or pretreatment before a surgical or cardiologic procedure. The effects of S-NO-HSA were tested at 2 h (T2) and 24 h (T3) after reperfusion.

The pressure–volume catheter inserted in the left ventricle permitted a real-time analysis of the LV hemodynamic performance. Through the Chart Software (AD Instruments), we measured LVEDP, EF, SV, dp/dt max, dp/dt min, max power, PAMP, and the isovolumetric relaxation time constant of the left ventricle tau-Weiss at the selected time points. In both pretreated and post-treated groups, the administration of S-NO-HSA demonstrated a protective effect on the left ventricle’s systolic and diastolic hemodynamic function, effectively preserving cardiac function against ischemia–reperfusion injury.

It is crucial to note the timing of drug administration in the post-treatment group (comprising post-HSA and post-S-NO-HSA), where the drug was introduced after the onset of ischemia. Consequently, it could only reach the infarcted ventricular area following reperfusion. Conversely, in the pretreatment group, S-NO-HSA was administered 15 min before coronary occlusion, allowing it to act on the area at risk of ischemia before occlusion occurred. In the pretreatment group, the positive effects of S-NO-HSA administration were immediately evident at T1 on systolic and diastolic function.

In the post-treatment group (post-HSA and post-S-NO-HSA), drug infusion commenced 15 min after LAD ligature; at T1, significant differences between treated (post-S-NO-HSA) and untreated (post-HSA) rats were observed solely in preload adjusted maximal power and dp/dt min. It is clear that the infusion of S-NO-HSA following the onset of ischemia is hindered by LAD ligation, limiting its penetration into myocardial tissue. Following 2 h of reperfusion (T2), all analyzed parameters, except dp/dt max, exhibited significant improvement in S-NO-HSA-treated rats compared to those receiving HSA. The reperfusion period permits S-NO-HSA to reach the injured myocardial tissue and exert its beneficial effects by releasing NO.

At T3, systolic and diastolic impairment in all the LAD occlusion groups, including the HSA group, was attenuated after the long reperfusion period (24 h) with hemodynamic values close to the sham group. However, the effect of S-NO-HSA was still evident, especially for dp/dt min and tau-Weiss, which were still improved in S-NO-has-treated rats.

Concentrations in the low nmol/L range are sufficient to prevent eNOS uncoupling and excessive reactive oxygen species and ONOO^−^ formation upon reperfusion. Administration of nitrate and nitrite before I/R injury was associated with increased heart eNOS expression and blunting of ischemia-induced iNOS augmentation [31]. We considered and compared the differences between pretreatment and post-treatment groups, and it is evident that an early and more extended infusion permits better ventricular function and contractility recovery. S-NO-HSA administered before coronary occlusion can reach the area at risk of ischemia and maintain constitutive NO levels longer after coronary occlusion, acting precociously and slowing down the cascade of events typical of the ischemia–reperfusion damage. The effect of S-NO-HSA is due to the prevention of eNOS uncoupling, thereby reducing reactive oxygen and nitrogen species formation. In addition, S-NO-HSA, due to NO release, prevents the induction of iNOS [32].

To better investigate the potential role of S-NO-HSA, we analyzed snap-frozen samples of the left ventricle exposed to I/R damage. We investigated the high-energy phosphates of the tissue and S-NO-HSA’s role in energy charge preservation. The concentration of PCr in rats treated with S-NO-HSA was significantly elevated compared to those treated solely with HSA, underscoring the protective efficacy of the NO-donor drug against ischemia–reperfusion injury. The maintenance of PCr levels and myocardial tissue energy charge suggests that NO supplementation to ischemic tissue could sustain tissue energy consumption at levels similar to baseline, thereby mitigating energy demand. Following 2 h of reperfusion, the energy profiles of both pretreated and post-treated rats exhibited similarity. After 24 h, the two treated groups exhibited a typical rise in PCr concentration and other quantified phosphates, indicating the gradual restoration of cellular energy sources. Conversely, rats infused with HSA displayed reductions in all high-energy phosphates, possibly indicative of exacerbated tissue damage in the region affected by ischemia–reperfusion injury. Notably, due to the brief duration of ischemia (only 30 min), histological analyses to quantify the extent of apoptosis or cellular necrosis were deemed unnecessary.

Furthermore, we measured the content of GSH and GSSG to explore the oxidative-reductive impacts of I/R injury across the various groups under study. GSH is a vital antioxidant molecule that is fundamental in detoxifying reactive oxygen and nitrogen species. Through its potent antioxidant properties, GSH mitigates oxidative stress and contributes to restoring and preserving cardiac function in the face of ischemic insults and subsequent reperfusion. Thus, the protective role of GSH underscores its significance as a therapeutic target for combating oxidative damage and preserving cardiac health under pathological conditions characterized by oxidative stress [33]. 

Notably, in rats treated with S-NO-HSA, the GSH concentration closely mirrors that of the sham group. This implies that a smaller proportion of GSH underwent reactions with reactive oxygen species and other free radicals, indicated by conversion into GSSG. In addition, this suggests that the glutathione reductase can cope with the number of reactive oxygen species converting GSSG back to GSH. The administration of S-NO-HSA as a NO-donor drug thus diminishes the production of free radicals, potentially indicating reduced uncoupling of eNOS and decreased formation of O_2_^−^ and ONOO^−^. In accordance, the percentage of GSSG was significantly increased in the HSA groups and significantly reduced by S-NO-HSA treatment at both 2 h and 24 h reperfusion.

The data from our experiments substantiate our working hypothesis and the ongoing and intricate debate concerning the dual nature of NO. This molecule has been the subject of extensive scrutiny and debate due to its seemingly paradoxical effects in various pathological contexts. This dichotomy has sparked a longstanding debate within the scientific community regarding the net effect of NO in ischemic pathophysiology.

Our findings suggest that administering S-NO-HSA has a promising avenue for mitigating myocardial damage in ischemia–reperfusion injury. 

### Limitations

For this study, we utilized a total of 50 rats. However, due to the necessary subdivision of groups, each group consisted of only five rats. We acknowledge that the limited number of rats per group may impact the generalizability of our findings, but it was adequate to detect significant differences between treated and untreated rats.

Unfortunately, we did not have the possibility to determine NO levels in this study. Furthermore, the duration of infusion requires consideration: While infusion ceased after 30 min of reperfusion in all subjects, the pretreatment group received infusion for 75 min, whereas the post-treatment group received it for 45 min. Therefore, the observed beneficial effects may be influenced by both the timing of infusion initiation and its duration.

Following 24 h of reperfusion, we observed a partial recovery of left ventricular function in rats that received HSA infusion. However, this recovery may be attributed to the relatively brief ischemic period. It is plausible that a longer ischemic duration in this experimental model would provide further insights into the effects of S-NO-HSA infusion on ischemia–reperfusion injury.

Another limitation of our study is that we did not measure cytokine release as we only focused on energy charge conservation, oxidative stress, and hemodynamic function.

## 5. Conclusions

The use of an NO donor with the properties of S-NO-HSA can be helpful both in a cardiologic context with an acute coronary syndrome that needs early percutaneous urgent treatment and reperfusion as well as in cardiac surgery with programmed cardioplegic arrest and, thus, programmed ischemia–reperfusion damage.

In our study, S-NO-HSA enhanced left ventricular post-ischemic function in both pretreated and post-treated groups.

We observed significant improvements in left ventricular function and energy metabolism in rats treated with S-NO-HSA compared to controls through hemodynamic assessments and biochemical analyses. The results highlight the potential of NO supplementation by S-NO-HSA in reducing the severity of I/R injury and promoting cardiac recovery.

Furthermore, by preserving GSH levels and the GSSG/GSH ratio, NO mitigates oxidative stress-induced damage and endothelial dysfunction and protects against myocardial injury.

A comprehensive understanding of NO biology and NO bioavailability in the context of I/R injury is promising for the development of novel treatment strategies aimed at improving outcomes in patients with ischemic heart disease.

## Figures and Tables

**Table 1:** Identification of area at risk (AR), relation of mass of ischemic area to left ventricle mass (AR/LV), and relation of necrotic area to ischemic area at risk (IF/AR). In the 4 groups, the time of ischemia was the same, but the time of reperfusion was different.

GROUP	TREATMENT	AR (g)	AR/LV (%)	IF/AR (%)
1	Ischemia 30′	0.220 ± 0.04	41.3 ± 3.5	66.1 ± 3.6
2	Ischemia 30′ + Reperfusion 5′	0.210 ± 0.20	39.5 ± 1.2	65.3 ± 3.8
3	Ischemia 30′ + Reperfusion 15′	0.232 ± 0.18	43.2 ± 2.5	67.1 ± 4.1
4	Ischemia 30′ + Reperfusion 30′	0.218 ± 0.08	38.6 ± 3.2	63.1 ± 6.4

## Data Availability

The data obtained in this study are available upon request to the corresponding author. The data are not publicly available because research is still in progress. However, data will be publicly available after the end of all studies connected to this preliminary phase.

## References

[1] Ambrosio G., Tritto I. (1999). Reperfusion injury: Experimental evidence and clinical implications. Am. Heart J..

[2] Rosenkranz E.R., Buckberg G.D. (1983). Myocardial protection during surgical coronary reperfusion. J. Am. Coll. Cardiol..

[3] Kloner R.A. (1993). Does reperfusion injury exist in humans?. J. Am. Coll. Cardiol..

[4] Zhang Y., Bissing J.W., Xu L., Ryan A.J., Martin S.M., Miller F.J., Kregel K.C., Buettner G.R., E Kerber R. (2003). Nitric oxide synthase inhibitors decrease coronary sinus-free radical concentration and ameliorate Myocardial stunning in an ischemia-reperfusion model. J. Am. Coll. Cardiol..

[5] Tsao P.S., Aoki N., Lefer D.J., Johnson G., Lefer A.M. (1990). Time course of endothelial dysfunction and myocardial injury during myocardial ischemia and reperfusion in the cat. Circulation.

[6] Rungatscher A., Milani E., Covajes C., Hallström S., Gottin L., Guidi G.C., Luciani G.B., Faggian G. (2017). Blood transfusions may impair endothelium-dependent vasodilation during coronary artery bypass surgery. Microvasc. Res..

[7] Malinski T. (2005). Understanding Nitric Oxide Physiology in the Heart: A Nanomedical Approach. Am. J. Cardiol..

[8] Darra E., Rungatscher A., Carcereri de Prati A., Podesser B.K., Faggian G., Scarabelli T., Mazzucco A., Hallström S., Suzuki H. (2010). Dual modulation of nitric oxide production in the heart during ischaemia/reperfusion injury and inflammation. Thromb. Haemost..

[9] Schulz R., Kelm M., Heusch G. (2004). Nitric oxide in myocardial ischemia/reperfusion injury. Cardiovasc. Res..

[10] Pearson P.J., Lin P.J., Schaff H.V. (1992). Global myocardial ischemia and reperfusion impair endothelium dependent relaxation. A possible cause of vasospasm after cardiopulmonary bypass. J. Thorac. Cardiovasc. Surg..

[11] Lefer D.J. (1995). Myocardial protective actions of nitric oxide donors after myocardial ischemia and reperfusion. New Horiz..

[12] Tsikas D., Sandmann J., Luessen P., Savva A., Rossa S., Stichtenoth D.O., Frölich J.C. (2001). S-Transnitrosylation of albumin in human plasma and blood in vitro and in vivo in the rat. Biochim. Biophys. Acta.

[13] Hallström S., Gasser H., Neumayer C., Fügl A., Nanobashvili J., Jakubowski A., Huk I., Schlag G., Malinski T. (2002). S-nitroso human serum albumin treatment reduces ischemia/reperfusion injury in skeletal muscle via nitric oxide release. Circulation.

[14] Rungatscher A., Hallström S., Linardi D., Milani E., Gasser H., Podesser B.K., Scarabelli T.M., Luciani G.B., Faggian G. (2015). S-nitroso human serum albumin attenuates pulmonary hypertension, improves right ventricular–arterial coupling, and reduces oxidative stress in a chronic right ventricle volume overload model. J. Heart Lung. Transplant..

[15] Dworschak M., Franz M., Hallström S., Semsroth S., Gasser H., Haisjackl M., Podesser B.K., Malinski T. (2004). S-nitroso human serum albumin improves oxygen metabolism during reperfusion after severe myocardial ischemia. Pharmacology.

[16] Hallström S., Franz M., Gasser H., Vodrazka M., Semsroth S., Losert U.M., Haisjackl M., Podesser B.K., Malinski T. (2008). S-nitroso human serum albumin reduces ischaemia/reperfusion injury in the pig heart after unprotected warm ischaemia. Cardiovasc. Res..

[17] Pelzmann B., Hallström S., Schaffer P., Lang P., Nadlinger K., Birkmayer G.D., Vrecko K., Reibnegger G., Koidl B. (2003). NADH supplementation decreases pinacidil-primed I K ATP in ventricular cardiomyocytes by increasing intracellular ATP. Br. J. Pharmacol..

[18] Fürst W., Hallström S. (1992). Simultaneous determination of myocardial nucleotides, nucleosides, purine bases and creatine phosphate by ion-pair high-performance liquid chromatography. J. Chromatogr..

[19] Lakritz J., Plopper C.G., Buckpitt A.R. (1997). Validated high-performance liquid chromatography: Electrochemical method for determination of glutathione and glutathione disulfide in small tissue samples. Anal. Biochem..

[20] Colasanti M., Suzuky H. (2000). The dual personality of NO. Trends Pharmacol. Sci..

[21] Parrino P.E., Laubach V.E., Gaughen J.R., Shockey K.S., Wattsman T.-A., King R.C., Tribble C.G., Kron I.L. (1998). Inhibition of inducible nitric oxide synthase after myocardial ischemia increases coronary flow. Ann. Thorac. Surg..

[22] Vinten-Johansen J., Zhao Z.-Q., Nakamura M., Jordan J.E., Ronson R.S., Thourani V.H., Guyton R.A. (1999). Nitric oxide and the vascular endothelium in myocardial ischemia-reperfusion injury. Ann. N. Y. Acad. Sci..

[23] Folino A., Losano G., Rastaldo R. (2013). Balance of nitric oxide and reactive oxygen species in myocardial reperfusion injury and protection. J. Cardiovasc. Pharmacol..

[24] Jhones S.P. (1999). Myocardial ischemia-reperfusion injury is exacerbated in absence of endothelial cell NOS. Am. J. Physiol..

[25] Amrani M., Gray C.C., Yacoub M.H. (1997). The effect of L-arginine on myocardial recovery after cardioplegic arrest and ischemia under moderate and deep hypothermia. Circulation.

[26] Hu H., Yamagishi T. (1997). Direct inhibition pf L-type Ca channels by S-nitrosothiol NO donors. Circ. Res..

[27] Totzeck M., Hendgen-Cotta U.B., Rassaf T. (2017). Nitrite-Nitric Oxide Signaling and Cardioprotection. Adv. Exp. Med. Biol..

[28] Hou J., He H., Huang S., Qian M., Wang J., Tan X., Han G., Song Y., Xu Z., Liu Y. (2019). A mitochondria-targeted nitric oxide donor triggered by superoxide radical to alleviate myocardial ischemia/reperfusion injury. Chem. Commun. (Camb.).

[29] Linardi D., Mani R., Murari A., Dolci S., Mannino L., Decimo I., Tessari M., Martinazzi S., Gottin L., Luciani G.B. (2022). Nitric Oxide in Selective Cerebral Perfusion Could Enhance Neuroprotection During Aortic Arch Surgery. Front. Cardiovasc. Med..

[30] Kamenshchikov N.O., Diakova M.L., Podoksenov Y.K., Churilina E.A., Rebrova T.Y., Akhmedov S.D., Maslov L.N., Mukhomedzyanov A.V., Kim E.B., Tokareva E.S. (2024). Potential Mechanisms for Organoprotective Effects of Exogenous Nitric Oxide in an Experimental Study. Biomedicines.

[31] Yassaghi Y., Jeddi S., Yousefzadeh N., Kashfi K., Ghasemi A. (2023). Long-term inorganic nitrate administration protects against myocardial ischemia-reperfusion injury in female rats. BMC Cardiovasc. Disord..

[32] Jakubowski A., Maksimovich N., Olszanecki R., Gebska A., Gasser H., Podesser B.K., Hallström S., Chlopicki S. (2009). S-nitroso human serum albumin given after LPS challenge reduces acute lung injury and prolongs survival in a rat model of endotoxemia. Naunyn-Schmiedeberg’s Arch. Pharmacol..

[33] Cheung P.Y., Wang W., Schulz R. (2000). Glutathione protects against myocardial ischemia-reperfusion injury by detoxifying peroxynitrite. J. Mol. Cell Cardiol..

